# Cross-laboratory evaluation of multiplex bead assays including independent common reference standards for immunological monitoring of observational and interventional human studies

**DOI:** 10.1371/journal.pone.0201205

**Published:** 2018-09-04

**Authors:** Krista E. van Meijgaarden, Bhagwati Khatri, Steven G. Smith, Anne M. F. H. Drittij, Roelof A. de Paus, Jelle J. Goeman, Mei M. Ho, Hazel M. Dockrell, Helen McShane, Simone A. Joosten, Tom H. M. Ottenhoff

**Affiliations:** 1 Department of Infectious Diseases, Leiden University Medical Center, Leiden, The Netherlands; 2 Bacteriology Division, Medicines and Healthcare Products Regulatory Agency-National Institute for Biological Standards and Controls, South Mimms, Potters Bar, Hertfordshire, United Kingdom; 3 Department of Immunology and Infection, London School of Hygiene and Tropical Medicine, Keppel Street, London, United Kingdom; 4 Department of Medical Statistics and Bioinformatics, Leiden University Medical Center, Leiden, The Netherlands; 5 The Jenner Institute, University of Oxford, Oxford, United Kingdom; California State University Fresno, UNITED STATES

## Abstract

**Background:**

Multiplex assays are increasingly applied to analyze multicomponent signatures of human immune responses, including the dynamics of cytokine and chemokine production, in observational as well as interventional studies following treatment or vaccination. However, relatively limited information is available on the performance of the different available multiplex kits, and comparative evaluations addressing this important issue are lacking.

**Study design:**

To fill this knowledge gap we performed a technical comparison of multiplex bead assays from 4 manufacturers, each represented by 3 different lots, and with the assays performed by 3 different laboratories. To cross compare kits directly, spiked samples, biological samples and a newly made reference standard were included in all assays. Analyses were performed on 324 standard curves to allow for evaluation of the quality of the standard curves and the subsequent interpretation of biological specimens.

**Results:**

Manufacturer was the factor which contributed most to the observed variation whereas variation in lots, laboratory or type of detection reagent contributed minimally. Inclusion of a common reference standard allowed us to overcome observed differences in cytokine and chemokine levels between manufacturers.

**Conclusions:**

We strongly recommend using multiplex assays from the same manufacturer within a single study and across studies that are likely to compare results in a quantitative manner. Incorporation of common reference standards, and application of the same analysis method in assays can overcome many analytical biases and thus could bridge comparison of independent immune profiling (e.g. vaccine immunogenicity) studies. With these recommendations taken into account, the multiplex bead assays performed as described here are useful tools in capturing complex human immune-signatures in observational and interventional studies.

## Introduction

Multiplex bead assays are commonly used for monitoring of complex multicomponent signatures of human immune responses in observational as well as interventional studies, such as treatment or vaccination trials. Measurement of secreted inflammatory mediators is relevant to many fields of study, including human infectious diseases, autoimmune diseases, cell signaling, neuroscience, cardiovascular diseases and cancer. In addition, more reagents and assays are becoming available for other species such as mice, porcines, canines, rats and non-human primates. These advances will facilitate cross-species comparisons of multicomponent immune signatures.

There are many components of the host immune response that may contribute to these signatures, including T-cells, B-cells, NK cells, neutrophils and monocytes. Multiple cytokines and chemokines can be produced simultaneously and the magnitude and balance of these different mediators defines the functional response signature [1]]. Over the last decade various commercially available multiplex suspension bead assays have been developed and gradually improved towards more robust and user- friendly assays. For example, polystyrene beads have been replaced with magnetic beads resulting in increased accuracy and reproducibility [2]]. The number of available analytes in a single assay has been expanded to approximately 40 cytokines or chemokines. Because the demand for easy and robust multi-parameter assays is still increasing multiple vendors have started to produce and market these assays. In human vaccination studies, sample numbers and volumes are often limited, in particular when involving young children, and frequently involve longitudinal sampling such that assays are preferred that are high-throughput, able to handle small sample volumes and still provide multi-factorial signature data. Current commercial multiplex bead assays can be performed on as little as 25–50 μl of culture supernatant from stimulated PBMC cultures, (diluted) venous blood or serum/plasma, and enable analysis of a large number of analytes. Another major advantage of these assays is that samples can be collected and stored, thus allowing serial measurements of samples from individuals in a single assay, thereby limiting inter-assay variation and thus optimising detection of possibly subtle perturbations in cytokines, chemokines and other secreted analytes over time [3, 4]. In the past few years many studies have been performed in which multiplex assays were tested for accuracy and reproducibility by including spiked samples or WHO standards, and compared to single analyte assays like ELISA and ELISpot [5–8]. Moreover their performance was evaluated in combination with optimized stimulation protocols for PBMC samples [9]. Even though multiplex bead assays cannot identify the cellular source of any biomarker measured but the total concentration of a cytokine or chemokine in a given sample, they provide powerful tools for multicomponent analysis of immunologic responses, which can guide further in-depth exploratory research to define the potential cellular sources and cellular interactions involved.

For each of the three major infectious diseases, TB, HIV and malaria, human immune responses induced by vaccination, in particular new experimental vaccines under evaluation are complex and multifactorial. Therefore, monitoring vaccine-induced changes over time needs to be broad and include a variety of cytokines and chemokines, rather than preselected single markers. In malaria it has been shown that different stages of parasitic infection involve different cytokine patterns, and that the balance of the cytokines determines the control of infection and disease outcome [10]. In BCG vaccination studies, complex cytokine profiles such as those obtained by multiplex bead assays proved to be valuable tools to discriminate vaccine induced responses across different continents [11, 12]. For each of these three diseases, field studies and trials are complicated because of large group sizes, long follow-up times and frequently poor local infrastructure in affected endemic areas. In order to achieve sufficient power, studies are frequently run at multiple sites.

To search for immune correlates of protection, it would be even more informative if it were possible to perform head-to-head comparisons of responses induced by different vaccine candidates in relation to clinical outcome, e.g. protection or disease. At the moment, the best proximate is to harmonize vaccine study design as well as immunological monitoring to the highest degree possible. Harmonization of assays such as the multiplex bead assay would allow an unique opportunity to conduct comparative analysis of vaccine induced immune responses over sites and over different vaccination trials. However, to run harmonization optimally it is critical to identify the factors that are responsible for variation within an assay. Therefore, we have here conducted a technical comparative study of multiplex cytokine and chemokine assays between 3 laboratories, using the kits from 4 manufacturers and 3 different lots, testing the same samples, including spiked samples, biological samples and a newly made reference standard as key reagents to understand the assay variation and interdependence. We then determined the major components responsible for variation based on advanced unbiased statistical methods. The results described below provide recommendations for optimal use of multiplex bead assays across different laboratories and studies.

The EURIPRED consortium aimed to identify the major factors in these assays that might influence the results and limit cross comparability between studies. The aim was also to develop solutions and reagents to overcome variability to enhance cross study comparative data analysis and evaluation, without real head-to-head comparisons, thereby accelerating the development of vaccines for globally important pathogens including TB, malaria and HIV.

## Results

### Standard curve analysis as stage-gates for each analyte

**Fig 1:**
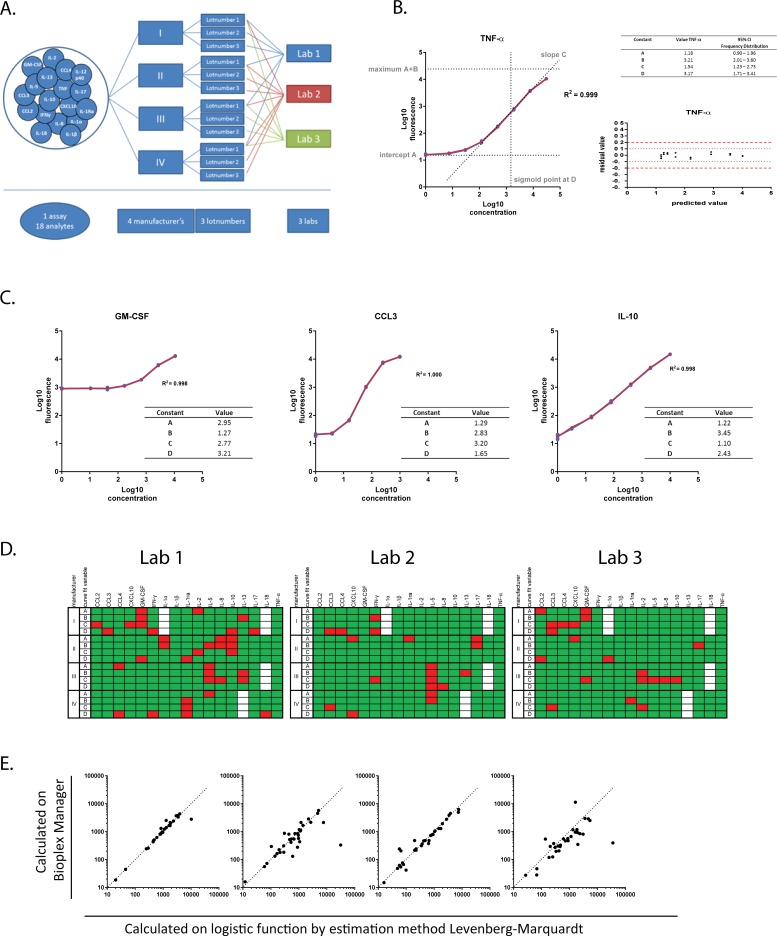
Description of standard curves by logistic function. (A) A schematic overview of all the variables tested is shown in Fig 1A. 17 Analytes (CCL2/MCP-1, CCL3/MIP-1 α, CCL4/MIP-1β, CXCL10/IP-10, GM-CSF, IFN-γ, IL-1 alpha, IL-1β, IL-10, IL-13, IL-17, IL-18, IL-1ra/IL-1F3, IL-2, IL-5, IL-8, TNF-α) out of the original 18 analytes of interest were assessed using kits from 4 different manufacturers; all the kits were tested in 3 independent laboratories. Each kit was tested from 3 different lot numbers, 1 lot was tested in all 3 laboratories whereas 2 additional lots were tested in a single laboratory (Lab).(B) Representative graph of the fluorescence vs the concentration of the standard curve shown in red. The curve is described by the logistic function f(x) = A + B/(1 + e−^C(x-D)^) where f(x) and x represents respectively the log transformed values of the fluorescence and the concentration. Here A is the intercept, A+B = maximum value of the curve, C is the slope of the curve, e is the natural logarithm base and D is the value of x at the sigmoid point shown here in red. The blue line shows the actual measured values. The residual vs the predicted fluorescence value is displayed in the insert. The predicted fluorescence value of each standard sample is calculated by the logistic function. The residual values represent the deviation of the measured fluorescence values from the predicted fluorescence values at all given concentrations.(C) Representative standard curves, originating from 2 labs, 2 manufacturers and 3 lots, are plotted for which the constant A (GM-CSF) and C (CCL3 and IL-10) are not within the 5–95% CI based on all 324 standard curves measured. (D)Summary of all standard curves analyzed at the 3 different labs with the same lot of reagents where green represents curve fitting for parameters A, B, C and D within the 5–95% CI and red indicates the parameters that do not meet the 5–95% CI criteria based on frequency distributions of 324 standard curves. Analytes that were not provided by the manufacturer in that specific assay are shown in white.(E)Correlation between analysis methods is plotted for each manufacturer. On the X-axis the values for the spiked samples of 500 and 1500 pg/ml as calculated by the estimation method according to Levenberg-Marquardt are represented against those calculated by the Bioplex software on the Y-axis.

Within the EC FP7 EURIPRED consortium (www.EURIPRED.eu) 18 cytokines and chemokines were selected commercially available multiplex bead assays were ordered for these analytes. Reagents/ kits were purchased from 3 different lots (batches) from each of the 4 different selected manufacturers (Bio-Rad, Millipore, Ebiosciences, R&D systems) (Fig 1A). Each manufacturer provided three sets of kits from one lot, which were then sent to the 3 participating laboratories (labs) for data comparison using the same lot when tested by each of the 3 labs (Fig 1A). In addition, 2 more lots from each manufacturer were tested. Each lab tested additional lots from a different manufacturer, to minimize bias towards possibly confounding lab specific parameters (Fig 1A). In addition, a third party universal detection reagent was included as an extra condition in all assays in all labs, in order to be able to compare all kits, using identical fluorescent detection reagents. Finally, all labs tested exactly the same spiked samples, reference standard and biological samples, with all kits and lots. In total 20 kits were tested in this study allowing analysis of 324 standard curves.

All data files from all manufacturers’ specific standard curves were collected and data were analyzed using a logistic function to describe the main characteristics of the standard curves. All data were analyzed using the same methodology, irrespective of instructions by the manufacturer. IL-12-p40 was excluded for comparative analyses as reagents for this cytokine were only available from one manufacturer. The logistic function for calculating the standard curve (red) showed a near perfect fit with a R^2^ of 0.999 to the actual data points measured (in blue) illustrated in Fig 1B, for TNF-α. Utilizing this logistic function the standard curves were described based on intercept A, maximum value A+B, slope C and sigmoid point D. The deviation of the measured fluorescence value versus the predicted value for the curve, was also calculated and plotted as residual value (right panel, Fig 1B). Since TNF-α has an excellent curve fit residual values are minimal.

All 324 manufacturers’ specific standard curves were analyzed for all 4 curve fitting parameters and the frequency distributions were plotted (S1A Fig). The 5–95% confidence intervals (CI) were used to classify the results (in Fig 1D). The parameters A, B, C and D were important indicators for the quality of the standard curves, in addition to the typically used R^2^. Plotting these parameters for frequency distribution for assays from the individual manufacturers resulted in unique profiles (S1B Fig). In particular, the slope of the standard curves for manufacturers III and IV and the sigmoid point for assays using reagents from manufacturer III deviated from the mean of the total curves analyzed. Each manufacturer provided its own standard, and parameters A to D allowed us to evaluate the quality of these curves and therefore the plausibility of the interpolated unknowns.

Fig 1C illustrates manufacturers’ specific standard curves with excellent R^2^ values for curve fitting, 0.998 for GM-CSF and IL-10 and 1.000 for CCL3, but these curves were nevertheless not ideal for interpolation and subsequent interpretation of test samples. The GM-CSF plot shows a value for intercept A that exceeds the 5–95% CI, indicating a high background value and a flat first part of the curve, making it difficult to interpret data in the lower range of the standard curve and resulting in a small dynamic range for interpolating data. For CCL3 and IL-10, both curves have a slope value outside the 5–95% CI. In case of CCL3 the value for the slope is high, which results in a very accurate concentration but a small dynamic range. For IL-10 the slope value C is low which suggests a limited accuracy in quantifying the unknown concentrations.

Evaluation of the quality of the standard curves was based on the score for each of the curve fit parameters for every analyte in each of the participating labs as shown in Fig 1D. Green indicates that the parameter fits within the 5–95% CI and red indicates failure to meet these criteria for that specific parameter. A total of 9.2% of the curve fitting parameters are outside the confidence interval criteria, and these were distributed over different kits and different labs. Only the assays for CCL3 from manufacturer I and for IL-5 and IL-8 from manufacturer III performed poorly in all 3 labs for the parameters C (slope) and D (sigmoid point). Overall the differences between standard curves, labs and lots were minimal.

Furthermore, we analyzed spiked samples with the widely used and commercially available BioPlex Manager software and compared this to the logistic function by the estimation method of Levenberg-Marquardt. Fig 1E shows the correlation of both analysis methods for each of the manufacturers. Samples on the diagonal line were not influenced by the analysis method, but as can be seen for all manufacturers, there were samples that deviated to either the horizontal or the vertical axes. For manufacturer IV, all samples were either underrated by the BioPlex software or overrated by the logistic function whereas this was only the case for individual measurements with the other manufacturers. It is also clear that manufacturer I and III demonstrated the best correlation in this analysis. Thus, it is important to harmonize also on the analysis platform to be used when cross-comparing data.

### An unbiased analysis identifies manufacturer as a major factor contributing to variation

Spiked samples were generated at the National Institute for Biological Standards and Controls, UK (NIBSC) by combining all 18 chemokines and cytokines at a concentration of either 500 pg/ml or 1500 pg/ml each. These concentrations were chosen to be in the range of interest of the biological samples based on previous experience, and are expected to fall in the linear part of the standard curves for most analytes.

**Fig 2:**
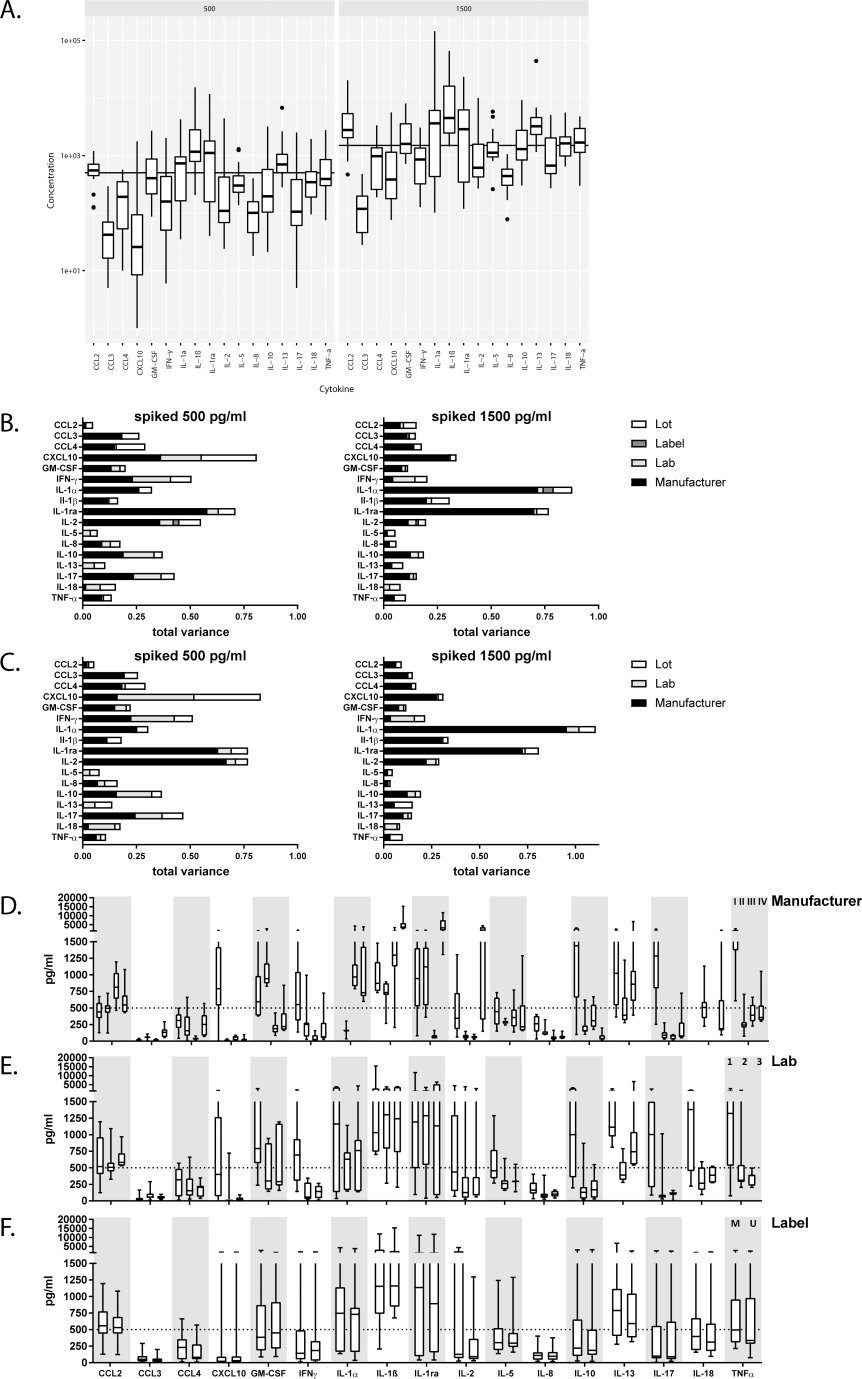
The commercial kit used is the main contributor of variance. (A)Left panel shows the concentration measured for all cytokines and chemokines using the spiked sample 500 pg/ml and the right part of the panel shows the results for spiked sample at 1500 pg/ml. Each box represents 40 data points. The line represents the expected concentration of each analyte. Boxes indicate the median and 25–75 percentile of data with whiskers at 1.5 times the interquartile range and dots indicating the extreme outliers above the 1.5 times interquartile range.(B)The bar graphs depict the contribution to the total variance of the different components, lot (white), detection label (dark grey), lab (light grey) and manufacturer (black) for the spiked sample 500 pg/ml on the left and spiked sample 1500 pg/ml on the right. (C) Same variance analysis as in B but with the universal PE detection label (D) The panel represents the 10 actual data-points for each analyte measured by the 3 labs for each of the manufacturers, I-IV from left to right, and all 3 lots and 2 detection labels. The dotted line indicates the reference value of 500 pg/ml. (E) The panel represents the data for the 500pg/ml spiked sample for each analyte over the different labs, for the same lot. The first box refers to lab 1, second to lab 2, third to lab 3 (total data-points for analysis for each lab n = 8). (F) Data for the variable detection label is plotted, the first box for each analyte indicates detection Streptavidin PE from the manufacturer (M) and the second box indicates the universal Streptavidin PE (U). Results for each analyte are analyzed for the 3 labs, 4 manufacturers and 3 kit lots (n = 20). All data points originate from the measurements of the 500 pg/ml spiked sample.

Fig 2A shows an unbiased Variance Component Estimation. This variance analysis weighs the contribution to total variance observed for every component of the assay independently. For all cytokines and chemokines measured in the spiked sample with a reference value of 500 pg/ml or 1500 pg/ml the boxes show the median and the 25–75% quantile of all data points, irrespective of lot, lab, manufacturer or the streptavidin-PE detection reagent (‘label’) used to detect the biotinylated detection antibodies (n = 40 per analyte; n = 20 for the manufacturer detection reagent plus another 20 data points for the universal detection reagent), with the whiskers showing the minimum and maximum values and the dots the extreme outliers above the 1.5 times interquartile range. Box-sizes are a measure of the total variance within the analyte, small boxes thus indicate that all kits yielded virtually the same results. Many analytes did not yield the expected concentrations and some deviated more than 2-fold. When averaged, only 6 analytes differed less than 25% for the spiked 500 pg/ml concentration, and 7 analytes for the spiked 1500 pg/ml concentration. All other analytes were more than 50% higher or lower than expected. All manufacturers performed equally poorly in this respect. For CCL4, CXCL10, IFNγ, IL-2, IL-10 and IL-17 the boxes for the 500 pg/ml spiked sample were larger than for the 1500 pg/ml spiked sample. This indicates that the 1500 pg/ml sample fitted the linear part of the standard curves better than the 500 pg/ml sample. For IL-1α and IL-1β the opposite is true, spiked samples with a concentration of 500 pg/ml gave less variation.

Fig 2B shows the contribution of each of the components (lot, lab, detection label or manufacturer) as part of the total variance. For nearly all analytes the manufacturer appears to be the largest parameter determining the overall variance. The other contributors to variance -lab, detection label and lot- are more subtle and their contribution to the total variance depends on the specific analyte. Overall the total variance for most analytes was limited and the different lots contributed only to a small proportion of the observed total variance. Streptavidin PE supplied by the manufacturer or universal streptavidin PE resulted in very similar values as can also be seen in the direct comparison in S2 Fig. If only data with the universal detection reagent are considered (Fig 2C), the total variance pattern hardly differs from that shown in Fig 2B, indicating that some factors other than the detection reagent determines variation between manufacturers. For the few analytes with very low total variances, such as IL-5, IL-13 and IL-18, the main component was not manufacturer but lab. Next to manufacturer, the remaining variation, e.g. between labs, then begins to have a relatively larger impact on the results. This can be seen in Fig 2B and 2C and also in Fig 2D, where we show the actual raw data that were used in the variance analysis.

In Fig 2D–2F, we plotted the different components of manufacturer, lab, and detection labels per analyte respectively. For some analytes, such as CCL4, CXCL10, IL-1α, IL-17 or TNF-α, one manufacturer was clearly different from the other 3, whereas for most analytes values were more scattered across the manufacturers. For the analytes that resulted in very small total variances, e.g. IL-5, IL-8, IL-13 and IL-18, the variance remained mainly explained by the lab-to-lab variation (Fig 2E), with minimal contributions of the manufacturer component. For CCL4, IL-1β and IL-2, that showed large variations between manufacturers, the lab-to-lab contribution to the overall variation was minimal. Finally, for all analytes the variation associated with the detection label, streptavidin PE, was marginal (Fig 2F).

### Results from biological samples are comparable within different multiplex manufacturers

Biologically relevant samples covering a wide range of stimulation induced responses were generated as described in the Materials and Methods. Supernatants were shared between labs and tested by all kits allocated to each particular lab.

**Fig 3:**
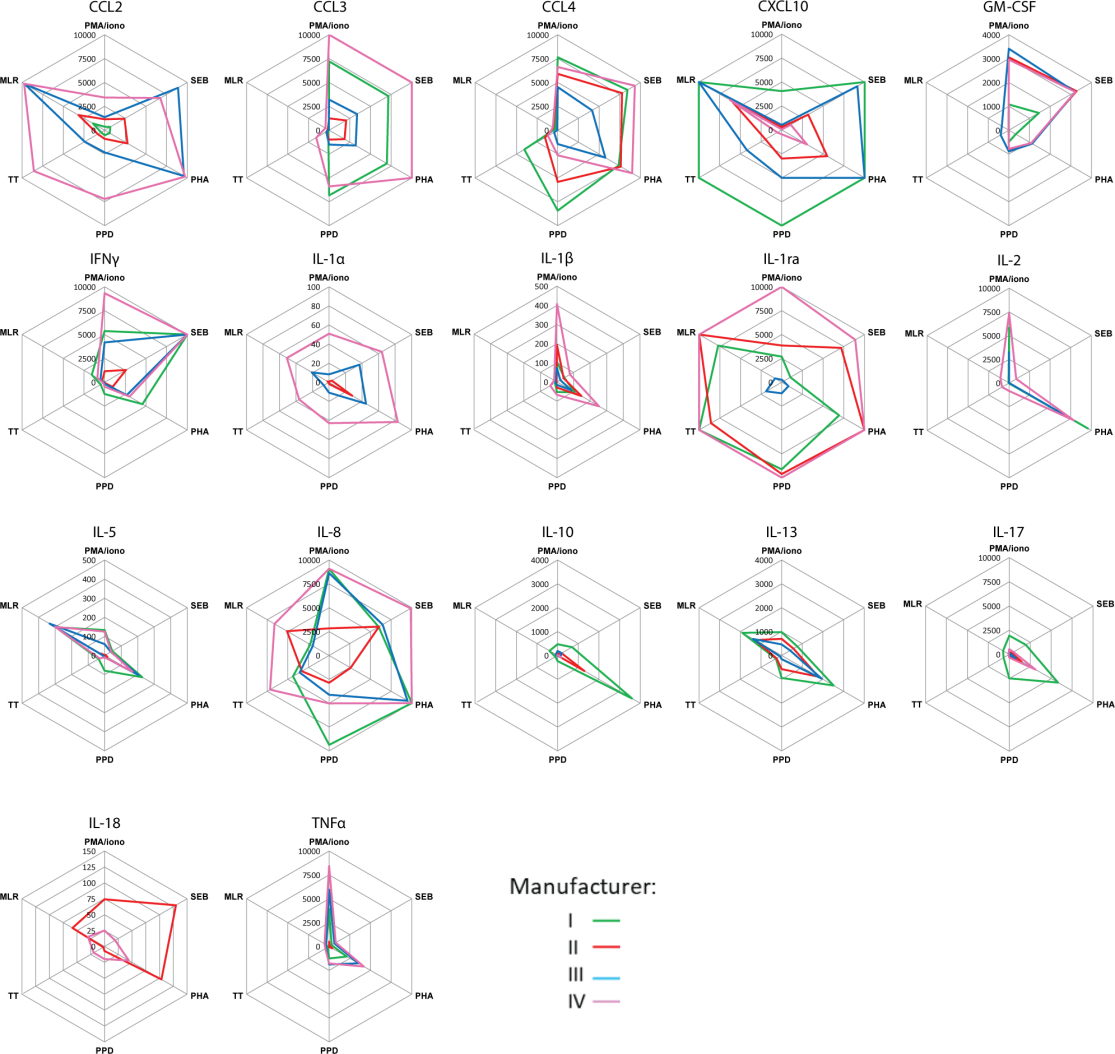
Ranking of biological samples is similar using kits from the different manufacturers. For each analyte and biological sample the data obtained with kits from the different manufacturers I (green), II (red), III (blue) and IV (pink) was plotted in a radar or spider plot with the stimuli at the corners of the plot. The geometric mean of all data-points (n = 5) for results from the different labs and kit lots is shown per sample using the manufacturer’s standard values and detection label. Results above the standard curve were set at 10 000 pg/ml.

Radar or spider plots were constructed to visualize the results from these samples in Fig 3. Specific stimuli are indicated in the corners of the plots, connecting lines are drawn between results obtained with the same kit, and each manufacturer is indicated by a different color. Using these plots, samples can be ordered or ranked based on the concentrations of analytes measured within each kit. If all lines follow the same pattern for a given analyte, the samples will give the same relative results even if the different samples do not have the same absolute concentrations. By contrast, crossing lines indicate qualitatively different types of responses for that particular analyte in those samples, suggesting that the ranking of these results is not comparable between the different manufacturers or studies. In general, most lines followed similar patterns and therefore, the relative (or ranked) response measured in these biological samples was similar (Fig 3), with the exception of an occasional crossing of lines at higher concentrations of analytes e.g. CCL2, IL-1ra or IL-8.

### A reference standard allows direct data comparisons

**Fig 4:**
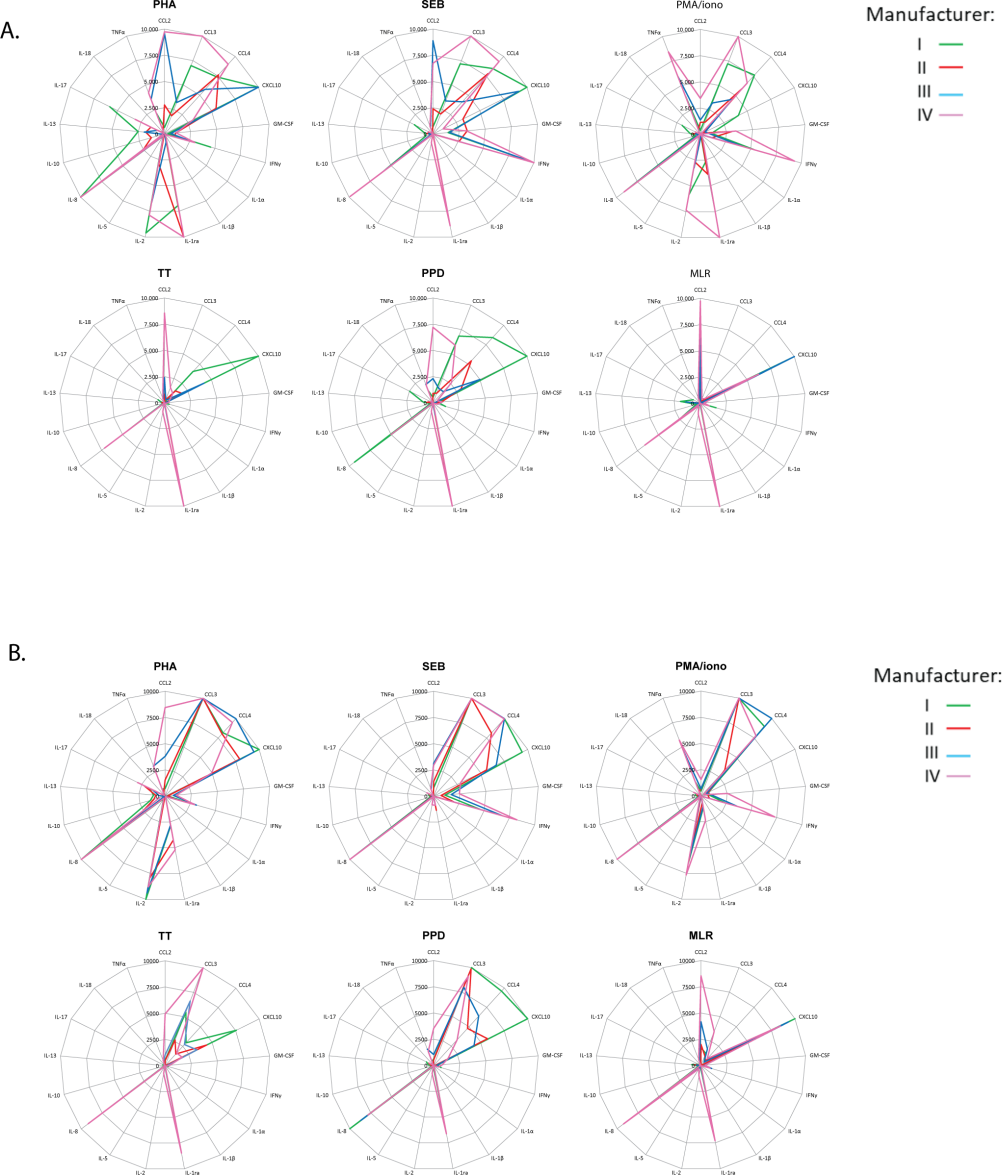
Common reference standard improved comparability for biological samples. (A) The results for the total cytokine/chemokine levels measured in the biological samples was plotted as geometric mean (n = 5) for the different labs and for the different lots. The kits from different manufacturers are shown in green (I), red (II), blue (III) and pink (IV) and data was related to the manufacturer’s standard values.(B) Results for the total cytokine/chemokine levels measured in the biological samples after re-analysis against the common reference standard curve.

As an alternative to ranking cytokines and chemokines across biological samples, data can also be plotted as cytokine and chemokine concentration per stimulation. Fig 4A represents spider plots for each of the stimulation conditions comparing the different cytokines and chemokines. Depending on the kit selected for the analysis, the results differ, and the relative ranking of cytokine and chemokine responses was completely altered. Since supernatants were identical, this is due to the variation in cytokine and chemokine concentrations measured with different kits (Fig 4A).

These variations could reflect truly different measurements but may also be the result of differences in data analysis methodology and extrapolation of values between the manufacturers. To distinguish between these two possibilities, we decided to assess whether variation in the observed concentrations between the different manufacturers could result from differences in the standard curves provided with each of the kits. An external, reference standard, based on serial dilutions from a spiked sample at 10,000 pg/ml generated at NIBSC, was incorporated in all assays, permitting calculation of unknown concentrations based on this reference standard. Concentrations in the biological samples were then calculated relative to this reference standard (Fig 4B). The reference standard equalized the concentrations and normalized the data to comparable patterns over the different analytes, between the different kits. These results demonstrate that incorporation of a reference standard overcame differences between kits obtained from manufacturers, thereby facilitating comparative analyses.

## Discussion

In this study we present the results of a technical comparative evaluation of multiplex cytokine and chemokine bead assays from 4 different manufacturers, with 3 lots from each manufacturer, and 2 detection reagents performed by 3 independent laboratories. These multiplex assays calculate analyte concentrations using standard curves. Standard curves must be evaluated by preset criteria for intercept, maximum value, slope and sigmoid point. Our variance component estimation identified the major component of variance as being the manufacturer, and that this could be largely overcome by inclusion of a common reference standard. Our recommendations for optimal use of multiplex bead assays across different laboratories and studies should enhance cross study comparative data analysis and evaluation, which should help accelerate the development of vaccines for globally important pathogens such as TB, HIV and malaria.

In head-to-head comparisons attention should be paid to harmonize not only the multiplex read out, but in addition preparation of samples and stimulations assays should be carefully harmonized. Synchronization between different studies and laboratories should include blood collection tubes, processing of whole blood or PBMCs. Furthermore, standard operation procedures for stimulation should be in place, describing cell numbers, media and type and concentration of stimulation reagents. Harmonized stimulation procedures in combination with the optimized multiplex bead array protocol should permit comparative analysis of cytokines responses between studies.

Inter- and extrapolation of test sample values are typically based on the standard curve, therefore understanding the characteristics and limitations of standard curves is of major importance, and should be incorporated in all data analyses and validation strategies. Standard curves were described here by their slope, intercept and sigmoid point regardless of the maximum concentration that was included in the standard curve. Maximum concentrations varied between manufacturers, affecting the detection range of individual kits. A standard curve with a small concentration range but a large fluorescence detection window may reflect a more accurate assay, but for sample screening purposes larger concentration ranges are preferred, particularly as samples are commonly used at a single dilution. As shown in Fig 1C, slopes and intercepts have great impact on the dynamic range, sensitivity and accuracy that can be achieved with a given assay. Standard curves should be assessed before data is inter- or extrapolated, and if one or more characteristics fail to fall within the 5–95% CI criteria, data should be interpreted with caution or discarded. Concentrations resulting from suboptimal standard curves can only be used to classify the measurements as being low, medium or high but no actual concentration can be calculated for these samples. Furthermore as shown in Fig 1E, the analysis method matters and we would recommend using the same analysis method especially when evaluating across different studies.

The variation observed in this study was largely the result of using kits from different manufacturers, while kit lots and the labs performing the assays contributed only a minor part of the variation. Also the universal detection reagent did not reduce variation significantly and was not a critical factor. Surprisingly, detection of spiked samples with fixed, known concentrations resulted in a large range of concentrations using the different kits, which deviated considerably from the calculated input amount. Amounts in the spiked samples were calculated against the standard curves provided with the kits. Formally variations in the amount of protein in either spiked sample or standard from the kit cannot be excluded. However, the large variation in values detected in the spiked samples between kits from different manufacturers may be caused by the fact that each manufacturer uses its own monoclonal or polyclonal antibody pair to detect the analyte, which may differ in affinity. Detailed information on antibodies in the kits is typically not disclosed by the manufacturers but it is likely that this is one of the major factors leading to the differences between the kits [13–15]. Although the absolute concentrations in the stimulated samples were different, the relative distributions were similar, and differences were abrogated by the use of a common reference standard. This suggests that the standards employed by the different manufacturers are also different and that these make a major contribution to the variations observed.

Detection of the spiked sample depends on the standard curve characteristics, in particular the maximum value measured in the standard curve. Some kits included a large working range from 3–10,000 pg/ml where the spiked samples fitted the ideal linear part of the curve perfectly, whereas others had tenfold lower ranges, from 3–1000 pg/ml. Hence, the spiked sample might be on the higher or lower end of the sigmoid area of the curve where the sensitivity and accuracy are less optimal. However, spiked samples were tested at 2 concentrations, at least one of which was expected to be in the linear part of the standard curve. Biological samples are expected to yield most reliable and reproducible results when in the linear part of the standard curve, and therefore in general, standard curves with the longest linear ranges will give most reproducible results. Thus replacing the kit standard with an universal standard could in particular enhance the results for analytes that have short-range standard curves in the original kits.

Besides the above mentioned manufacturer’s specific differences, the use of buffers and matrix diluents differs per assay. It has been reported by Jager et al [16] that proteins other than antibodies can interfere with the assay. Since we performed the assays according to manufacturer’s instructions we could not investigate potential effects of buffers and matrix diluents. However, if these would have been a major factor we would have expected all results from a particular manufacturer to deviate systematically in the same direction, rather than analyte specific differences as observed here. In addition, data extrapolation with the common reference standard would not have normalized the data as well as it did. Furthermore we did not take into account the time span that could be present between experiments in real-life, as our assays were performed within the limited timeframe of six months. Generally in clinical studies the evaluation of immune responses will only be initiated after the sample collection is completed, and by analyzing all samples per individual in one occasion, testing all samples within a limited time span and using one assay lot. However, if multiple assays need to be performed over longer periods of time with different kit lots, we believe there is an added value of including a reference standard or spiked samples.

In vaccine development and immune monitoring studies, many have exploited the advantages of the multiplex bead assay as a versatile screening tool suitable for small sample volumes and large sample numbers [2, 11, 12, 17]. At the same time, many laboratories have also encountered inconsistencies in the determined absolute values, discrepancies between technical specifications and the actual performance, differences in sample preparations and differences in the methodology for executing the various assays [2, 6, 14, 18]. Breen et al.[15]reported that the multiplex assay may not be sufficiently reproducible for repeated determination of absolute cytokine concentrations but may be very useful in longitudinal studies where relative values are more interesting. However, we suggest that the inclusion of a common reference standard could rectify this. To better comprehend the observed differences in absolute values Khan et al.[6] have compared the absolute concentrations (pg/ml) with the complementary international units (IU), but the differences remained. Nechansky et al.[5] included WHO standards in their assays displaying an overestimation of the results based on the underestimation of the standard curves in the kits compared to these WHO standards. These differences in absolute values of the multiplex bead assays remain problematic in head-to-head comparisons when different assays are used. Based on our conclusions that the manufacturer is the key component responsible for variance, and that the lab performing the assay and the kit lot are the minor variants in the assays’ variability. We suggest that multisite comparisons are feasible as long as longitudinal studies are measured in the same assay with the same kit from the same manufacturer, and when external reference standards are included.

In this study, we conducted a thorough analysis on the characteristics and comparability of the same highly standardized commercially available multiplex beads assays over 3 different labs, 3 different lots and with kits from 4 different manufacturers. To compare as many possible conditions that will be of greatest value to the fields of immune profiling, we undertook costly test conditions with duplicate reference standards, which might not be feasible in real life. However, we suggest that adding a reference standard, or one or more spiked samples are important for data comparisons when analyzing clinical trials and larger cohorts in which such extra costs are relatively marginal and quantitative outcomes extremely valuable. We suggest that the kit for a particular manufacturer itself is not critical for standardising across the field. Our data show that the use of our proposed common standard is capable of overcoming the differences introduced by the choice of manufacturer. Furthermore it may not be possible to decide unambiguously for each research group and research question which would be the best manufacturer since this also depends on the sample source to be measured, e.g. sera or plasma, whole blood or PBMC culture supernatants and the analytes of interest. The sample source will also influence the expected cytokine and chemokine levels, and this impacts the choice of manufacturer and kit.

The range of the reference standard curve, replacement of the manufacturer’s standards, and the number of replicates used can be further discussed, and assays can be custom-made to fit specific needs and study design. Once these decisions are made we recommend that kits from one manufacturer are used for each separate study analysis, with common reference standards, one analysis method is applied and that standard curves are critically judged per cytokine before extrapolation of the data. This will allow the maximum information to be obtained on the biomarkers being analyzed and enable reproducible and robust bio-signatures to be identified. Multiplex bead arrays are thus a very useful tool to monitor human immune responses over time, such as during disease or following vaccination.

## Materials and methods

### Cytokine and chemokine multiplex beads assays

The following cytokines and chemokines were selected based on solicited interests of researchers in the field of poverty related diseases, covering a wide array of Th1, Th2, Th17 as well as monocytic responses; CCL2/MCP-1, CCL3/MIP-1 α, CCL4/MIP-1β, CXCL10/IP-10, GM-CSF, IFN-γ, IL-1 α, IL-1β, IL-10, IL-12 p40, IL-13, IL-17, IL-18, IL-1ra/IL-1F3, IL-2, IL-5, IL-8, TNF-α.

All multiplex magnetic bead assays were performed according to manufacturer’s protocols. Assays included in this study were (I) Biorad (Bio-Plex pro Human Cytokine 27-plex assay, Veenendaal, The Netherlands; 15 analytes were analyzed per kit), (II) Ebioscience (ProcartaPlex human Cytokine & Chemokine panel 1A, 34-plex, Hatfield, United Kingdom; 17 analytes were analyzed per kit), (III) Merck Millipore (Milliplex Human Cytokine/chemokine magnetic bead premixed 29-plex kit, Watford, United Kingdom; 17 analytes were analyzed per kit) and (IV) R&D systems (Human Magnetic Luminex Screening assay 16-plex, Biotechne, Abingdon, United Kingdom; 16 analytes were analyzed per kit). Some manufacturers supplied quality control samples with the kits, but since not all kits provided those and they only indicated an expected range, they were not included. Manufacturer specific standards and the common reference standards were run in duplicate, spiked samples and biological samples were run as single measurements. Detection of samples was performed with the Streptavidin-PE detection label supplied by the manufacturer and a duplicate set of samples was analyzed with a universal Streptavidin-PE (Becton Dickinson, Eerbodemgem, Belgium). All laboratories used the BioPlex 100 system for data acquisition.

### Preparation of spiked samples and reference standard

Eighteen cytokines were selected for the preparation of spiked samples and reference standard to cover a broad range of immunological responses. Out of 18 cytokines; 5 cytokines were WHO International Standard from NIBSC—GM-CSF (Cat. No. 88/646), IL-1α (Cat. No. 86/632), IL-1β (Cat. No. 86/680), IL-8 (Cat. No. 89/520) and TNF-α (Cat. No. 88/786); 5 cytokines were WHO reference reagents from NIBSC–IL-10 (Cat. No. 93/722), IL-13 (Cat. No. 94/622), IL-17 (Cat. No. 01/420), IL-18 (Cat. No. 03/200) and IL-5 (Cat. No. 90/586), and IL-1ra/IL-1F3 (Cat. No. 92/644) from NIBSC is a non-WHO reference reagents. The rest of the 7 cytokines were obtained from commercial suppliers–IFN-γ (Cat. No. 14-8319-80) and CCL4 (Cat. No. 14-8938-80) from Ebioscience; IL-2 (Cat. No. 11011456001) from Sigma; CCL2/MCP-1 (Cat. No. 279-MC-010), hIL-12p40 (Cat. No. MAB6091-100) and CXCL10 (Cat. No. 266-IP) from R&D systems; CCL3 (Cat. No. 582802) from Biolegend. For preparing reference standard, all 18 cytokines were reconstituted as per instruction for use or manufacturers’ recommendations, further diluted with PBS+0.1% BSA and mixed to obtain final concentration of 10,000pg/ml of each analyte at NIBSC. For preparing spiked samples, 18 cytokines were mixed and diluted with PBS+0.1%BSA to obtain 500pg/ml and 1500pg/ml of each analyte.

### Preparation of biological samples

For preparation of the biological samples PBMC were isolated using Ficoll density centrifugation (LUMC Pharmacy, Leiden, the Netherlands) from buffy coats of healthy blood bank donors (Sanquin, Amsterdam, The Netherlands). PBMCs were cultured at 6x10^6^ cells/ml in T75 flasks in RPMI supplemented with glutamine (Gibco, Thermo Fisher Scientific, Bleiswijk, the Netherlands) and 10% FBS (Hyclone, GE Healthcare Life Sciences, Eindhoven, the Netherlands), in the presence of the following stimuli: PMA (50 ng/ml) and ionomycin (250 ng/ml) (both Sigma Aldrich, Zwijndrecht, the Netherlands) for 24 hours, PHA (5μg/ml, Remel, Thermo Fisher Scientific, Bleiswijk, The Netherlands) for 3 days and SEB (2 μg/ml, Toxin Technology, Sarasota, FL, USA), PPD for in vitro use (5 μg/ml, Serum Statens Institute, Copenhagen, Denmark) and Tetanus Toxoid (RIVM, The Netherlands) for 5 days. A mixed lymphocyte reaction sample was generated by mixing PBMCs from two random donors (1:1) and culturing them for 6 days at a concentration of 3x10^6^ cells/ml from each donor. At the end of the cultures, supernatants were harvested, aliquoted and lyophilized for distribution and use in the multiplex bead assays. No unstimulated control supernatants were included and cytokine and chemokine levels of the biological samples were thus not background corrected. The PMA/ionomycin, PHA, SEB and MLR stimulated supernatants were diluted 5x before measurements. All labs reconstituted and diluted the lyophilized samples in the same way and tested the same dilutions.

### Statistical analysis

All data were analyzed by SPSS (IBM statistics v23) where the estimation method of Levenberg-Marquardt was used with the logistic formula f(x) = A + B/(1 + e−^C(x-D)^) to perform the non-linear regressions describing the standard curves. In total 324 out of 325 standard curves were analyzed originating from 20 kits with 15 to 17 analytes per kit as stated by the description of the multiplex bead assays. One standard curve was excluded due to a technical issue. All standard curves included 6 serial dilution points in duplicate. Besides the logistic function BioPlex Manager software (v6.1) was used for correlation of both analysis methods. Variance componence analysis [19] was performed per cytokine with variance components for manufacturer, lot, lab and detection label using R, a language and environment for statistical computing (**R** Foundation for Statistical Computing; 2014, Vienna, Austria) and the script included in the S1 File. The box and whisker plots indicate the 25–75% quantile, with the median at 50% and the lower whisker equal to the smallest observation greater than or equal to the lower hinge -1.5*IQR. The upper whisker equals the largest observation less than or equal to upper hinge + 1.5 * IQR. Outliers are plotted as dots. Data was plotted using Graphpad Prism v7.0 and Microsoft Excel 2010.

## Supporting information

S1 Fig Frequency distribution analysis of the curve fit parameters. (A) The frequency distribution and statistics for the parameters A (intercept), B (maximum), C (slope) and D (sigmoid point) of all 324 standard curves analyzed. (B) Breakdown of the different parameters A, B, C and D over the different manufacturer’s (I-IV) is shown as frequency analyses with dotted lines at median and 5–95% CI.
(PPTX)

S2 Fig Comparison of the component ‘detection label’ does not show significant differences in variance. For the spiked samples (500 and 1500 pg/ml) the variance of the manufacturer label (M) were compared to that using the universal detection label (U). The left graph shows the data for spiked samples at 500 pg/ml and the right graph for the 1500 pg/ml concentration.
(PPTX)

S1 File R script. (DOCX)

S2 File Raw data. (XLSX)
