# Supplementary figures and images for: Cross-laboratory evaluation of multiplex bead assays including independent common reference standards for immunological monitoring of observational and interventional human studies
Source: PLoS One. 2018 Sep 4;13(9):e0201205. doi: 10.1371/journal.pone.0201205 (PMC6122788; doi:10.1371/journal.pone.0201205)

## Slide 1
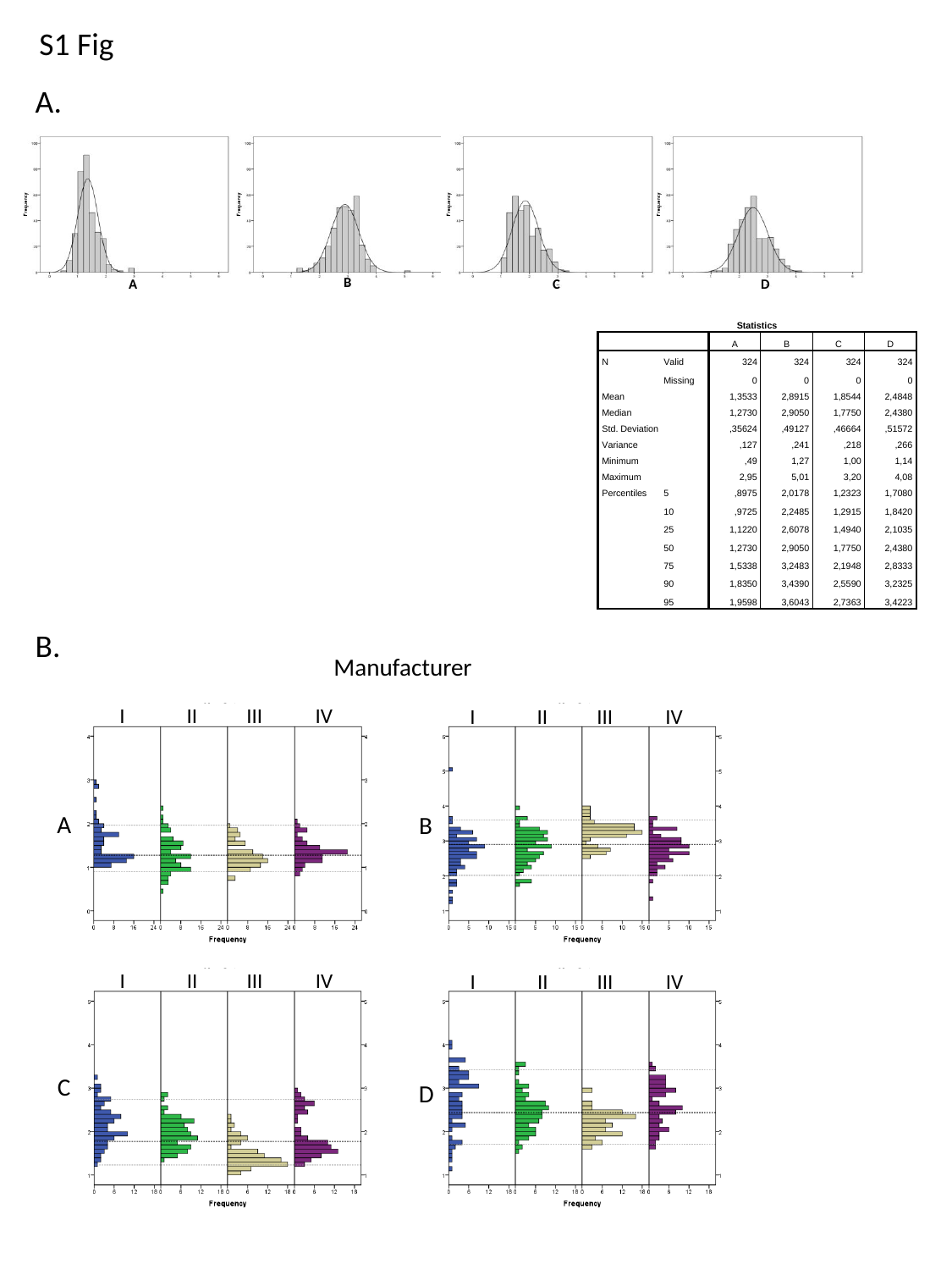

S1 Fig
A.
B
A
C
D
B.
Manufacturer
I
II
III
IV
I
II
III
IV
A
B
I
II
III
IV
I
II
III
IV
C
D

Supplement: S1 Fig — (A) The frequency distribution and statistics for the parameters A (intercept), B (maximum), C (slope) and D (sigmoid point) of all 324 standard curves analyzed. (B) Breakdown of the different parameters A, B, C and D over the different manufacturer’s (I-IV) is shown as frequency analyses with dotted lines at median and 5–95% CI. (PPTX) [file pone.0201205.s001.pptx]

## Slide 1
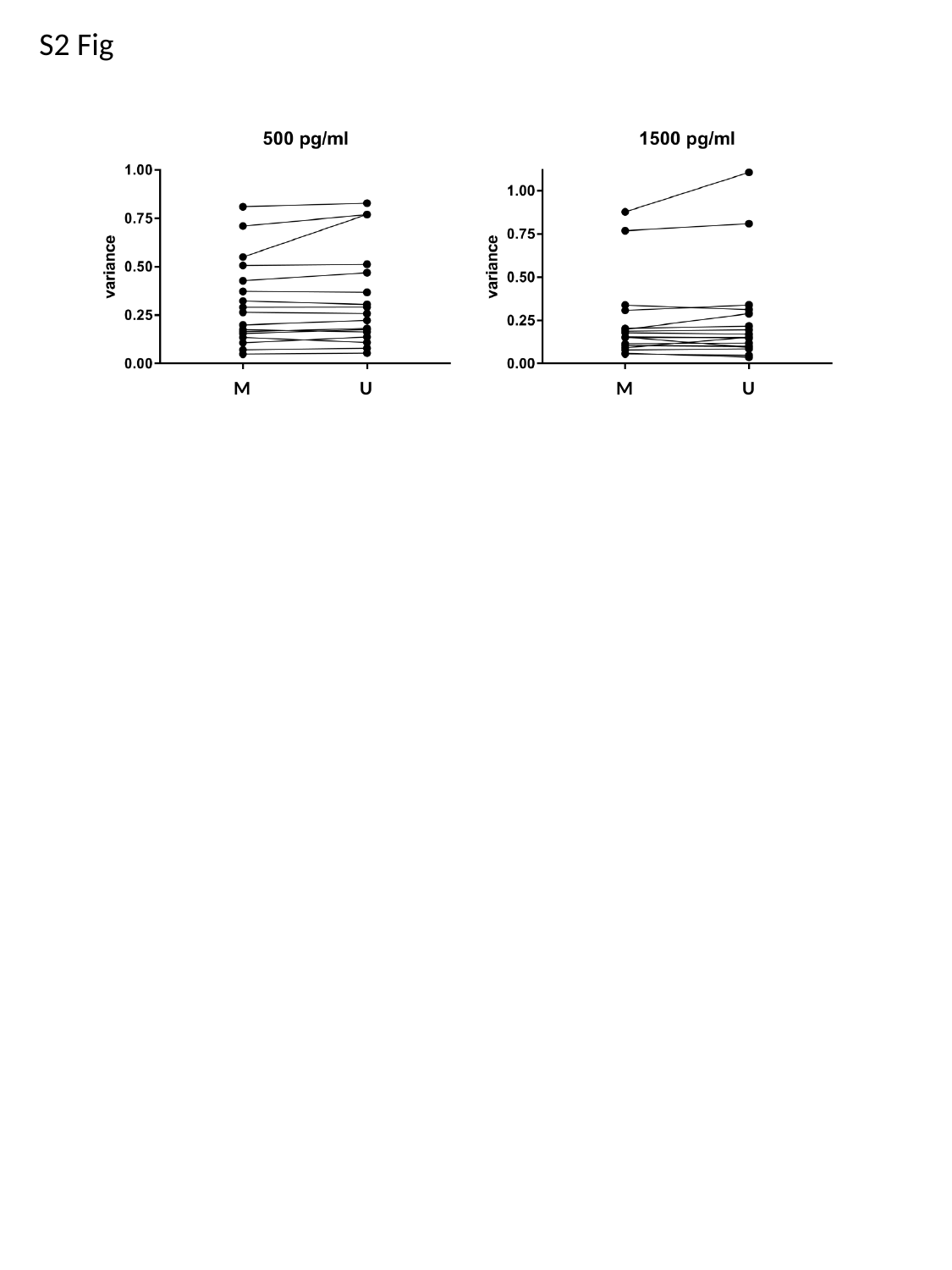

S2 Fig
M
U
M
U

Supplement: S2 Fig — For the spiked samples (500 and 1500 pg/ml) the variance of the manufacturer label (M) were compared to that using the universal detection label (U). The left graph shows the data for spiked samples at 500 pg/ml and the right graph for the 1500 pg/ml concentration. (PPTX) [file pone.0201205.s002.pptx]
