# Supplementary material for: Cross-laboratory evaluation of multiplex bead assays including independent common reference standards for immunological monitoring of observational and interventional human studies
Source: PLoS One. 2018 Sep 4;13(9):e0201205. doi: 10.1371/journal.pone.0201205 (PMC6122788; doi:10.1371/journal.pone.0201205)
Supplement: S1 File — (DOCX) [file pone.0201205.s003.docx]

**R Script**

library(varComp)

varcomps <- matrix(0, nlevels(D$means), 4)

ests <- numeric(nlevels(D$means))

rownames(varcomps) <- names(ests) <- levels(D$means)

colnames(varcomps) <- c('Manufacturer', 'Lab', 'Label', 'residual')

for (i in 1:nlevels(D$means)) {

print(i)

thismean <- levels(D$means)[[i]]

thisD <- D[D$means==thismean,]

if (length(unique(thisD$Manufacturer))==1) {

res <- varComp(log10(Concentration) ~ 1, data=thisD,

random = ~ Lab + Label)

print(i)

varcomps[i,] <- c(NA, res$varComps, res$sigma2)

} else {

res <- varComp(log10(Concentration) ~ 1, data=thisD,

random = ~ Manufacturer + Lab + Label)

varcomps[i,] <- c(res$varComps, res$sigma2) }

ests[i] <- 10^res$fixef}

varcomps.perc <- varcomps/rowSums(varcomps)

100*round(varcomps.perc, 4)

res <- data.frame(100*round(varcomps.perc, 4), variance=rowSums(varcomps))

write.table(100*round(varcomps.perc, 2), file='varcomps.txt', quote=FALSE, sep='\t', col.names=NA)

write.table(res, file='varcomps_2.txt', quote=FALSE, sep='\t', col.names=NA)
